# *Camellia japonica* Root Extract Increases Antioxidant Genes by Induction of NRF2 in HeLa Cells

**DOI:** 10.3390/plants11212914

**Published:** 2022-10-29

**Authors:** Jung-Hwan Kim, Heejung Yang, Kee K. Kim

**Affiliations:** 1 Department of Pharmacology, School of Medicine, Institute of Health Sciences, Gyeongsang National University, Jinju 52727, Korea; 2 Laboratory of Natural Products Chemistry, College of Pharmacy, Kangwon National University, Chuncheon 24341, Korea; 3 Department of Biochemistry, College of Natural Sciences, Chungnam National University, Daejeon 34134, Korea

**Keywords:** NRF2, *Camellia japonica* L., antioxidant

## Abstract

*Camellia japonica* L. (Theaceae) has been used for medicinal and cosmetic purposes in East Asian countries. Most functional components were obtained from the upper parts of the tree, such as leaves, flowers, or seeds. Here, we report a functional effect of the 80% methanolic extract of *C. japonica* root (CJRE) on antioxidative stress in HeLa cells. The nuclear factor erythroid-derived 2-related factor 2 (NRF2) is a key transcription factor that triggers the induction of oxidative stress-relating genes and drug detoxification. As result, CJRE showed a strong anti-radical scavenging effect in a dose-dependent manner. In addition, the induction of antioxidant response elements (ARE)-luciferase activity was maximized at CJRE 200 µg/mL. Furthermore, CJRE induced the mRNA levels of *HO-1* and *NQO1* by the nuclear NRF2 accumulation. As a possible mechanism of Nrf2 activation, the phosphorylation of p38 and ERK1/2 signaling might fortify the NRF2 induction as well as its stability. However, the phosphorylation of AKT is rather decreased. Taken together, CJRE may potentiate the antioxidant effects by increasing the NRF2 signaling through MAP kinase signaling and the properties of its radical scavenging activity. Thus, CJRE could apply for other medicinal and cosmetic purposes.

## 1. Introduction

Nuclear factor erythroid 2-related factor 2 (NRF2), a transcription factor, is considered a master key for controlling oxidative stress by the induction of antioxidant-related genes and drug detoxification. The functional role of NRF2 has been considered critical for oxidative stress-derived pathophysiological outcomes, such as aging, inflammation, neurodegenerative disorders, metabolic syndromes, and numerous cancers [1,2,3,4].

Currently, it is known that the level of NRF2 can be controlled by the proper degradation machinery. In normal conditions, KEAP1 kelch-like ECH-associated protein 1(KEAP1) takes part in the NRF2 degradation in the presence of the ubiquitinoylation process. However, if once stabilized by triggering factors, such as oxidative stress, electrophilic stress, or various natural chemicals [5,6,7,8,9,10,11,12], translocated NRF2 can stimulate the induction of NRF2 target genes.

Among the NRF2 target proteins, heme-oxygenase-1 (HO-1), NAD(P)H: quinine oxidoreductase-1 (NQO-1), and glutamate-cysteine ligase catalytic (GCLC) were reported as the representative proteins. In addition, the induction of these genes is regulated by NRF2/antioxidant response elements (AREs) in their promoter regions [13,14]. 

The functional activation or inhibition of NRF2 has been widely studied. As indicated above, KEAP1 is a representative NRF2 inhibiting factor by degrading the protein via ubiquitin-dependent proteasomal degradation [15]. On the other hand, mitogen-activated protein kinases (MAPKs), such as p38 and ERK1/2, activated protein kinase α (AMPKα) [16,17], AKT, protein kinase C (PKC)cyclic-AMP- [18,19,20,21], and IQGAP1 [6] are studied as a positive regulator of NRF2. 

Numerous naturally-occurring chemicals were screened as NRF2 inducers, many of which induce the NRF2 activation through various signaling pathways, involving mitogen-activated protein kinases (MAPKs), cAMP-activated protein kinase (AMPK), and AKT, as well as protection mechanisms of NRF2 from ubiquitinyl proteasomal degradation [22,23].

*Camellia japonica* L. (Theaceae) is an evergreen tree that can grow in East Asian countries, such as Korea, Japan, and China; it has been used for medical and cosmetic purposes. This is because the use of the tree has been focused on above-ground parts, such as flowers, leaves, seeds, stems, and fruits for different biological activities. Each part of the tree has been used for different purposes; for example, the flowers are used for the inhibition of porcine epidemic diarrhea virus (PEDV) replication and inhibition of melanogenesis [24,25,26,27]; the stems for anti-inflammatory and cytotoxic activities [28,29]; the seeds for the inhibition of ethanol absorption [30,31]; the fruits for the treatments of inflammation, gastric ulcers, and breast cancer [32,33]; and the leaves for antioxidants [34]. 

As previously, novel saponins were isolated from the root of *C. japonica* and showed an increase in the ARE-luciferase activity in the HeLa cells [35]. Here, we explored the potential antioxidative activity and NRF2 activity using the methanolic extract of *C. japonica* roots.

## 2. Results

### 2.1. The Chemical Profile of CJRE

In the previous study, we isolated eleven triterpenoidal saponins and evaluated ARE luciferase activities by Nrf2 accumulation in the nucleus [35]. The chemical profile of eleven triterpenoidal saponins in CJRE were analyzed by the retention times and mass fragment patterns with the responding chemical standards (Supplementary Figures S1 and S2). 

### 2.2. CJRE Increases NRF2 Activity through ARE System in HeLa Cells

**Figure 1:**
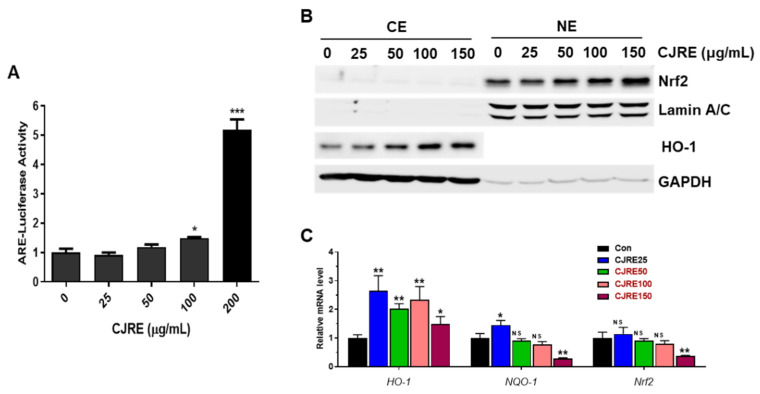
CJRE increases the NRF2 activity in HeLa cells. (**A**). ARE-luciferase activity was measured after treatment with various doses of CJRE for 12 h in HeLa cells. (**B**). The cells were treated with various doses of CJRE for 24 h and Western blotting was performed. (**C**). qPCR data showed the different mRNA levels of *HO-1*, *NQO*-1, and *NRF2* after treatment with different doses of CJRE for 24 h. * *p* < 0.05; ** *p* < 0.001; *** *p* < 0.0001; NS, not significant.

**Figure 2:**
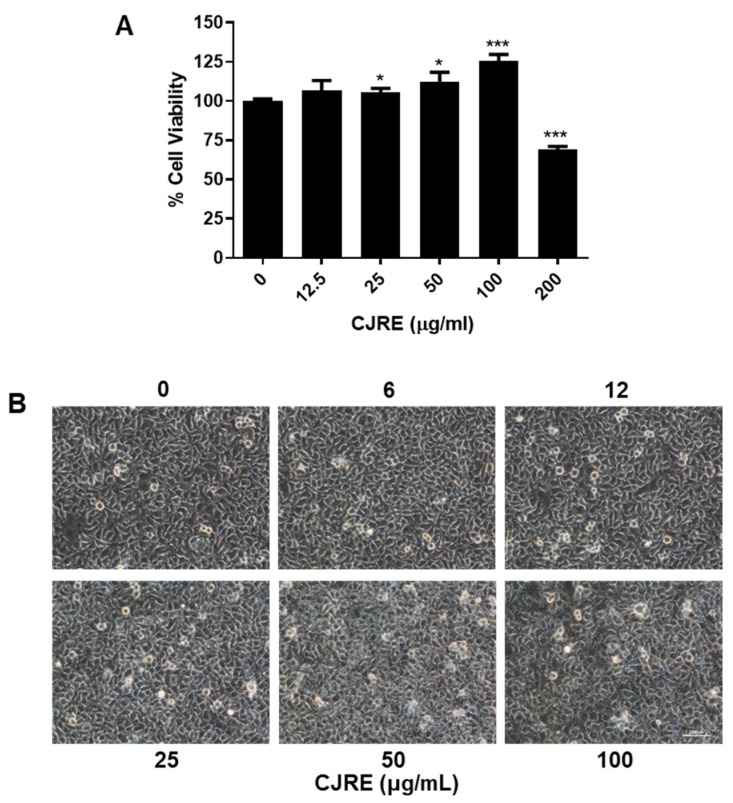
Cytotoxicity of CJRE on HeLa cells. (**A**). HeLa cells were treated with different doses of CJRE for 24 h and the cytotoxicity was measured using MTT assay. (**B**). HeLa cells were imaged under brightfield microscopy after treatment with different doses for 24 h. Scale bar, 250 µm; * *p* < 0.05; *** *p* < 0.0001.

To evaluate the NRF2 activity by CJRE, an ARE-luciferase assay was performed in HeLa cells. As result, CJRE (Figure 1A) dramatically increased the ARE-luciferase activity in a dose-dependent manner. Moreover, the nuclear accumulation of NRF2 was significantly increased at 150 μg/mL CJRE. In addition, HO-1 was also dramatically increased by the treatment with 150 μg/mL CJRE (Figure 1B) as expected. Likewise, qPCR results showed that the HO-1 mRNA level was increased by CJRE. As previously, we observed that a mild cytotoxic range of drugs might increase NRF2 activity; we measured the cytotoxic effect of CJRE. The result of the MTT assay indicated that CJRE showed a growth inhibitory effect at 200 μg/mL (Figure 2A). However, cell death was not shown at the highest concentration according to the cell images (Figure 2B). Thus, it suggests that CJRE triggers the NRF2 activity resulting in the inductions of NRF2 target genes.

### 2.3. CJRE Has a Radical Scavenging Activity

**Figure 3:**
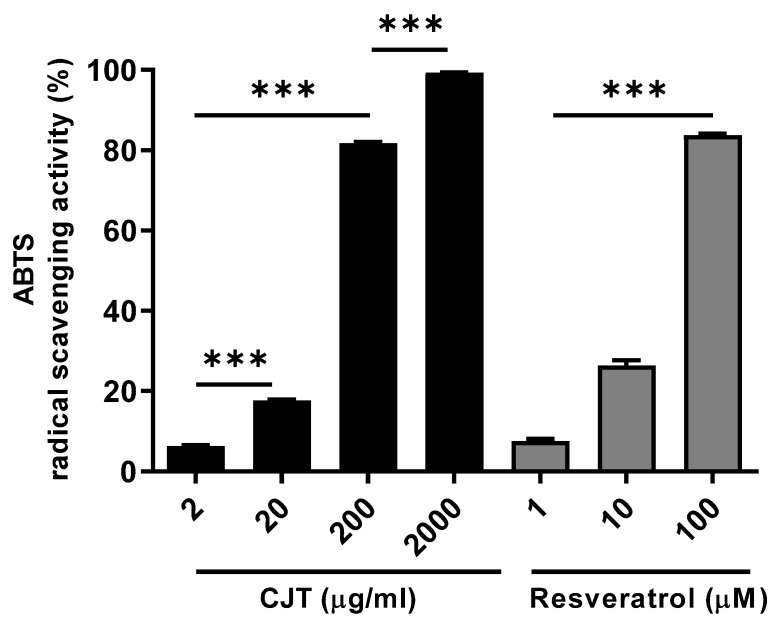
CJRE showed a strong antioxidative activity with dose-dependent manner. ABTS assay indicates that CJRE has a strong antioxidative effect. Resveratrol was used as the positive control. *** *p* < 0.0001.

To test the radical scavenging effect of CJRE, an ABTS test was performed. As result, radical scavenging activity was potentiated at 200–2000 μg/mL CJRE (Figure 3). 

### 2.4. CJRE Potentiates the NRF2 Stability in HeLa Cells

**Figure 4:**
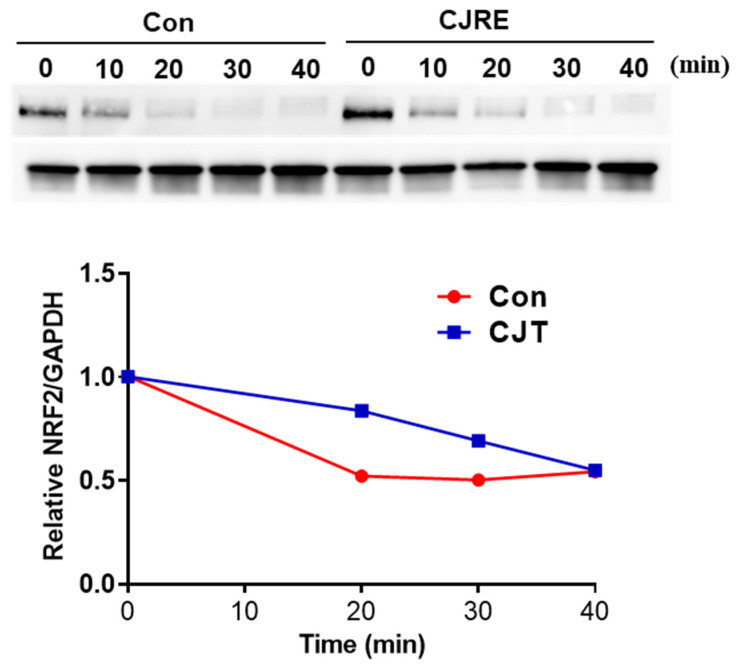
CJRE enhanced the NRF2 stability in HeLa cells. Cells were treated with CJRE (100 μg/mL) for 24 h and followed CHX (5 μg/mL) treatment for different time points. Next, the collected lysates were subjected to Western blotting. After densitometric analysis, the relative NRF2/GAPDH amounts were indicated in the graph (bottom panel).

To test whether CJRE affects NRF2 stability, the degradation of NRF2 was monitored by using cycloheximide (CHX). As a result, relatively the NRF2 protein was gradually degraded in 30 min by CJRE (100 μg/mL) when compared to the control (Figure 4).

### 2.5. CJRE Increases the Transcriptional Activity NRF2 Gene

**Figure 5:**
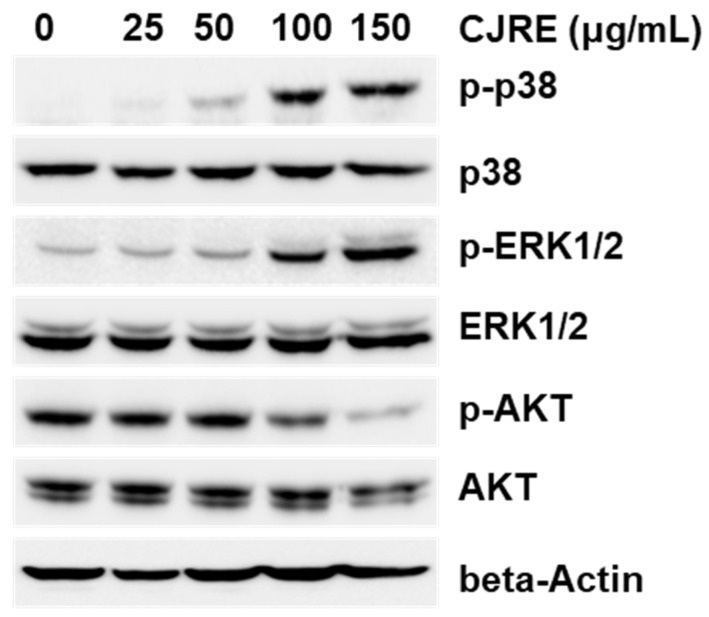
CJRE induced phosphorylation of p38 and ERK1/2 in HeLa cells. HeLa cells were treated with different doses of CJRE for 24 h, and whole cell lysates were subjected to Western blotting with indicated antibodies.

Because mitogen-activated protein kinases (MAPKs) and AKT could induce NRF2 as previously reported [39], we examined the effect of CJRE on MPAK activation. As a result, CJRE dramatically increased the phosphorylation of p38 and ERK1/2 in a dose-dependent manner within 24 h. However, AKT is rather dephosphorylated as we increased the CJRE doses. Thus, CJRE could trigger the induction of NRF2 via the activation of p38 and ERK1/2 (Figure 5).

## 3. Discussion

Because NRF2 plays an important role in many diseases in conjunction with oxidative stress, various studies have been investigated to find NRF2 activator using naturally-occurring substances. On the other hand, it might be a useful strategy to find molecules that inhibit the KEAP1-mediated NRF2 degradation pathway [15]. 

It is important to control the NRF2 before normal cells changed to abnormal because many cancerous cells already obtained the NRF2 activity to escape from the excessive oxidative insult. Thus, it might be beneficial to obtain the NRF2 activators as a concept of chemoprevention for many pathophysiological symptoms including cancers.

Here, we report the effect of CJRE on NRF2 activation with a possible molecular mechanism. Here, CJRE potentiated the increase of NRF2 in the nucleus, resulting in the induction of NRF2 target genes, such as HO-1 and NQO-1, via ARE motifs in HeLa cells; CJRE potentiated the increase of NRF2 in the nucleus, resulting in the induction of its target genes, such as HO-1 and NQO-1, via ARE motifs in HeLa cells. In our previous report, some saponins isolated from C. japonica roots showed weak NRF2 induction [35]; however, it is necessary to investigate NRF2 activity using crude extracts for other purposes.

In addition, it is important that CJRE exhibits a free radical scavenging effect. This could allow CJRE’s chemicals to directly control excess cellular free radicals. Therefore, multifunctional CJRE can easily protect cells.

To maintain the activity of NRF2, not only the signal transduction to NRF2, but also the stability of NRF2 is important. Likewise, a representative degradation system of NRF2 is proteasomal degradation by ubiquitination, as previously reported [23,39]. Thus, it is possible that CJRE could inhibit NRF2 degradation by inhibiting proteasomal degradation.

Regarding the mechanism of Nrf2 activation by CJRE, MAPKs, such as p38 and ERK1/2, might be involved, as previously reported [39]. This is because CJRE strongly increased the phosphorylation of ERK1/2 and p38, but not AKT. The basal level of the phosphorylation of AKT of HeLa cells was rather inhibited by CJRE. Thus, inhibition of cell proliferation by CJRE might be involved in AKT signaling. Although the activation of MAPKs could be a direct signaling factor, the qPCR results showed that the mRNA level of NRF2 was not changed by CJRE. The signal might be for other signals because of the extract.

**Figure 6:**
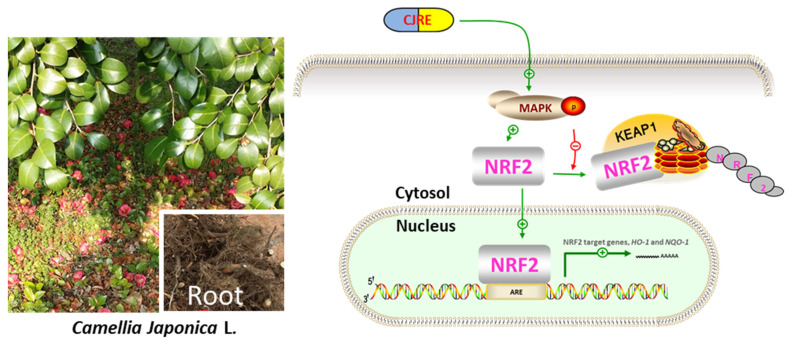
Scheme showing a possible role of CJRE on NRF2 signaling in HeLa cells. CJRE can activate the NRF2 signaling by phosphorylation of MAPK and inhibition of NRF2 degradation, resulting in the induction of NRF2 target genes, such as *HO-1* and *NQO-1.*

The difference between our study and other studies is the use of different parts of the tree; this is because a limited study was performed using the root of this tree. As previously, we reported that *C. japonica* roots contained many novel saponins that were not reported in other parts of the tree. Thus, it is necessary to study the function of bioactive chemicals from the root extract of the tree. Since *C. japonica* is considered not only for a good ornamental purpose, but also for medicinal usage, the application of this plant is wide due to the many bioactive ingredients. Here, we propose that CJRE can activate the NRF2 signaling by the activation of MAPK and inhibition of NRF2 degradation, resulting in the induction of antioxidant-relating genes (Figure 6). Thus, CJRE can apply for chemoprevention or cosmetic purposes as an NRF2 inducer. 

## 4. Materials and Methods 

### 4.1. Chemicals

*C. japonica* roots (CJRE) were collected in the southwest (Haenam, Jeollanam-do) of South Korea in August 2016. The dried *C. japonica* roots (11.5 kg) were chopped into 3–5 cm pieces and were extracted with 80% MeOH for 3 h twice using an ultrasonic apparatus. The methanolic extract of CJRE (476 g) was yielded after evaporating in vacuo and freeze-dried as powder. Anti-NRF2 (ab137550) and anti-HO-1 (ab68477) antibodies were obtained from Abcam (Cambridge, MA). Antibodies against phospho-AKT (#4060), and AKT (#4691), phospho-p38(#9211), p38 (#9212), phospho-pERK1/2 (#4377), pERK1/2 (#4696), were purchased from Cell Signaling Technology (Danvers, MA). Antibodies against Lamin A/C, GFP, beta-Actin, and GAPDH (sc-25778) were obtained from Santa Cruz Biotechnology (Santa Cruz, CA, USA).

### 4.2. Cell Culture

HeLa cells (American Type Culture Collection (ATCC, Manassas, VA, USA)) were cultured in DMEM medium (10% fetal bovine serum, antibiotic–antimycotic (100 units/mL of penicillin, 100 µg/mL of streptomycin, and 0.25 µg/mL of amphotericin B)) in a humidified incubator (37 °C, 5% CO_2_, and 95% air). Cells were grown at 60–70% confluency for sub-culturing and all the experiments.

### 4.3. LC-qTOFMS Analysis

Ten milligrams of the lyophilized samples were resolved in 100% MeOH (5 mg/mL) and filtered using a 0.2-μm cellulose membrane. For LC-qTOFMS analysis, the sample was injected on a Xevo-G2 qTOF mass spectrometer (Waters, Milford, MA, USA) connected with a Waters Acquity UPLC system. In total, 1 μL of the CJRE sample was analyzed under the gradient solvent condition from 10% to 90% of 0.1% formic acid in acetonitrile on the analytical column, a Waters Acquity BEH C18 column (150 mm × 2.1 mm, pore size 1.7 μm), for 15 min. The temperature of the column oven and the flow rate were set at 45 °C and 0.3 mL/min, respectively. The ESI conditions of the MS experiments were as follows: 2.0 kV capillary voltage, 35 V cone voltage, 100 °C source temperature, 250 °C desolvation temperature, 50 l/h cone gas flow, and 600 l/h of desolvation gas flow in the positive ion mode. Leucine enkephalin (*m/z* 554.2615 [M-H]^−^) was used to ensure the mass accuracy and reproducibility of the optimized MS conditions.

### 4.4. Cell Toxicity Assay

The cellular toxicity of CJRE was measured using a MTT assay in HeLa cells, as previously reported [36]. Briefly, cultured cells in 48-well plates were treated with different doses of CJRE (0–200 μg/mL) for 24 h. Then, MTT stock solution (20 μL from 5 mg/mL stock solution) was added to each well. After 1 h incubation, culture media were suctioned. Then, purple-colored formazan was dissolved with DMSO and measured at 570 nm (Varioskan^TM^ LUX, Thermo Scientific^TM^).

### 4.5. Radical Scavenging Test 

The antioxidant activity of CJRE was measured as previously described [37]. Briefly, 20 μL of CJRE at various concentrations was incubated with 80 µL of fresh-prepared ABTS^+^ (2,2’-azino-bis(3-ethylbenzothiazoline-6-sulfonic acid) radical solution for 4 min in the dark condition, and followed by an optical measurement at 650 nm using the EMax Plus Microplate Reader (Molecular Devices, San Jose, CA, USA). The scavenging activity was calculated as followed: ABTS^+^ radical scavenging activity (%) = [1 − (Abs_CJRE_ − Abs_blank_ of sample)/Abs_control_] × 100, where Abs_control_ indicates the absorbance of ABTS^+^ radical solution diluted in the water, Abs_CJRE_ indicates the the absorbance of ABTS^+^ radical solution mixed with CJRE, and Abs_blank_ stands for the absorbance of CJRE with distilled water. 

### 4.6. ARE Luciferase Assay

To evaluate NRF2 activity by CJRE, the ARE-luciferase activity was measured using the dual-luciferase reporter system (Promega). Briefly, HeLa cells cultured in 48-well plates were treated with different concentrations of CJRE (0–200 μg/mL) for 6 h after co-transfection with a pGL4.21_3×ARE plasmid (60 ng/well) [22] and a pRL-Renilla luciferase plasmid (20 ng/well) overnight. Then, 10 μL lysates were applied to the ARE luciferase activity. The Renilla luciferase activity was used to normalize the transfection.

### 4.7. Western Blot Analysis

HeLa cells were treated with different doses as indicated in the Figures. Nuclear/cytosolic proteins and whole-cell lysates were isolated with an M-PER buffer and a RIPA buffer, respectively. [38]. The protein concentration was determined with a BCA reagent (Thermo Scientific, Waltham, MA). Proteins (10–30 µg) were then separated on a gradient SDS-polyacrylamide gel (4–20%) and transferred onto a nitrocellulose membrane. After the membrane blocking process (5% non-fat dry milk in PBS, 0.1% Tween-20) for 1 h, the primary antibodies (1:1000) were incubated at 4 °C overnight. Protein signals were visualized using an ECL solution after incubation (1 h) with horseradish peroxide-conjugated secondary antibodies. 

### 4.8. Real-Time PCR Analysis 

Total RNA was purified using a TRIzol reagent (Invitrogen, Carlsbad, CA, USA) according to the manufacturer’s guides. Later, synthesized cDNA using the qScript cDNA Synthesis kit (QuantaBio, Beverly, MA, USA) were applied to qPCR. For the qPCR reaction, PerfeCTa SYBR Green FastMix (QuantaBio, Beverly, MA, USA) was used as the following conditions: initial step at 95 °C for 30 s; second step, 45 cycles at 95 °C for 5 s, 60 °C for 10 s; and cooling at 4 °C for 10 s using QuantStudio^TM^ 5 (Applied Biosystem^TM^). The following qPCR primer sets were used: *NRF2-F*, 5′-TCT TGC CTC CAA AGT ATG TCA A-3′ and *NRF2-R*, 5′-ACA CGG TCC ACA GCT CAT C-3′; *NQO-1-F*, 5′-TCC TTT CTT CTT CAA AGC CG-3′; and *NQO-R*, 5′-GGA CTG CAC CAG AGC CAT-3′; *HO-1-F*, 5′-GAG TGT AAG GAC CCA TCG GA-3′ and *HO-R*, 5′-GCC AGC AAC AAA GTG CAA G-3′; *GAPDH*-*F*, 5′-AAG GTG AAG GTC GGA GTC AA-3′ and *GAPDH-R*, 5′-AAT GAA GGG GTC ATT GAT GG-3′. 

### 4.9. Protein Stability Assay 

To evaluate the NRF2 stability by CJRE, cells were treated with CJRE (100 µg/mL) for 24 h and followed by treatment with cycloheximide (5 µg/mL) for 40 min at different times. Later, the NRF2 amount was visualized by Western blotting and quantified by densitometry.

### 4.10. Statistical Analysis

The results were presented as the mean ± SD. Statistical analysis was executed using a two-tailed Student’s *t*-test on the unpaired data using GraphPad Prism 8.4.3 software. *p* < 0.05 was considered statistically significant. 

## Data Availability

The data presented in this study are available upon request from the corresponding author.

## Supplementary Materials

The following supporting information can be downloaded at: https://www.mdpi.com/article/10.3390/plants11212914/s1, Figure S1: LC/MS chromatogram of CJRE; Figure S2: The MS spectral data and structures of eleven compounds (**1**–**11**) in CJRE.
