# Supplementary material for: Camellia japonica Root Extract Increases Antioxidant Genes by Induction of NRF2 in HeLa Cells
Source: Plants (Basel). 2022 Oct 29;11(21):2914. doi: 10.3390/plants11212914 (PMC9655997; doi:10.3390/plants11212914)
Supplement: Supplementary file 1 [file plants-11-02914-s001.zip › plants-1970330-supplementary.pdf]

Supplementary material

Camellia japonica root extract increases antioxidant genes by induction of NRF2 in HeLa cells

Jung-Hwan Kim[1*], Heejung Yang[2*], and Kee K. Kim[3]

[1]Department of Pharmacology, School of Medicine, Institute of Health Sciences, Gyeongsang National University, Jinju, 52727, Republic of Korea
[2]College of Pharmacy, Kangwon National University, Chuncheon 24341, Republic of Korea
[3]Department of Biochemistry, College of Natural Sciences, Chungnam National University, Daejeon, 34134, Republic of Korea

* To whom correspondence should be addressed.
Jung-Hwan Kim, Ph.D.
Tel: +82-55-8072
E-mail: junghwan.kim@gnu.ac.kr, (J.H. Kim)
Heejung Yang, Ph.D.
Tel: +82-33-250-6919.
E-mail: heejyang@kangwon.ac.kr, (H. Yang)

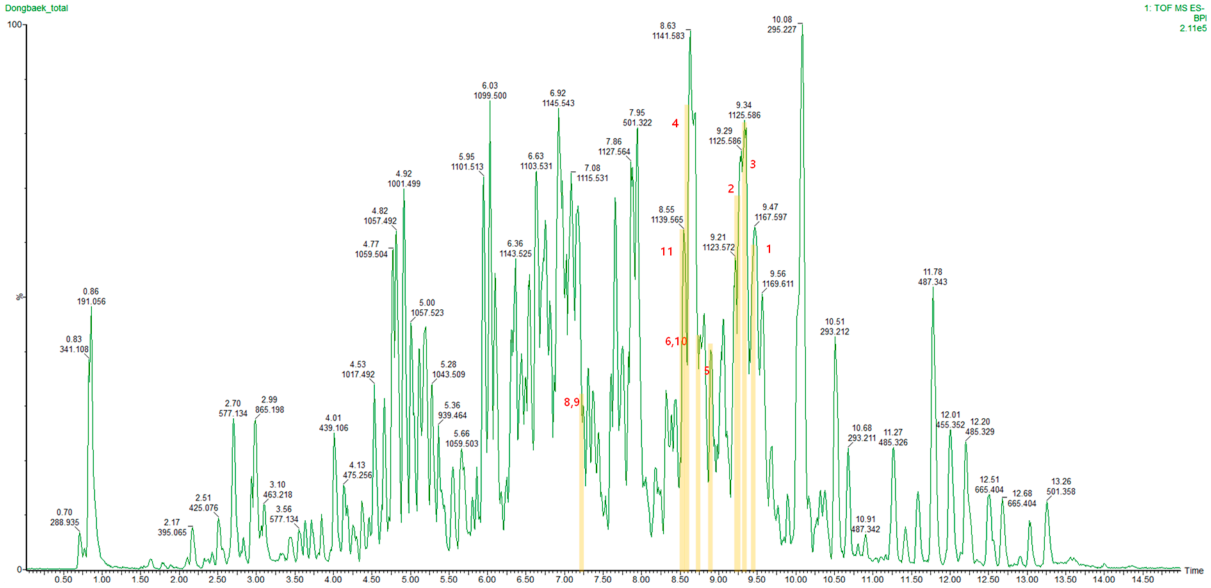

Supplementary Figure S1. LC/MS chromatogram of CJRE. A. Ten compounds were identified in the CJRE sample except for compound 7 due to the lower quantity in the sample. The structures and MS spectral data corresponding to eleven triterpenoidal saponins were listed in Supplementary Figure 2. The analytical conditions are described in Material and Methods.

Compound 1

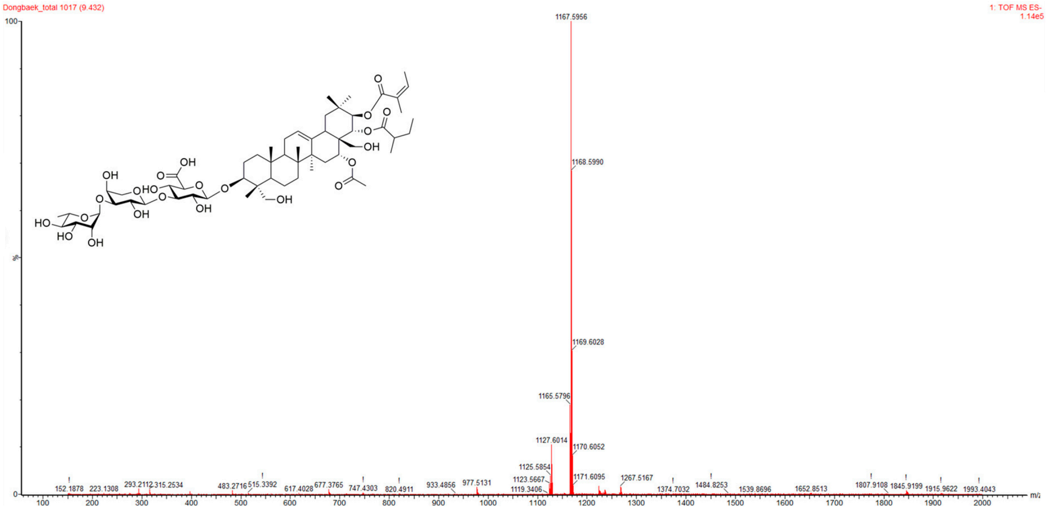

Compound 2

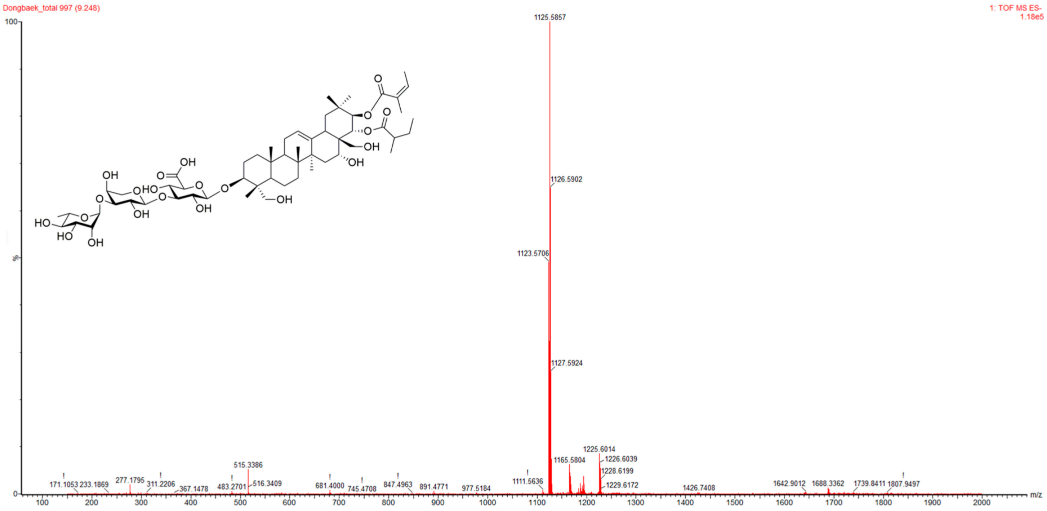

Compound 3

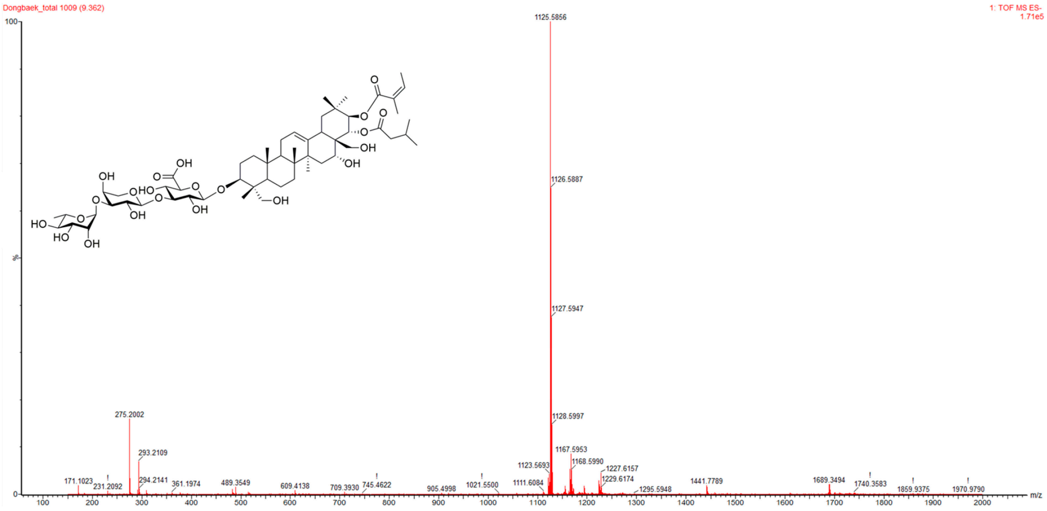

Compound 4

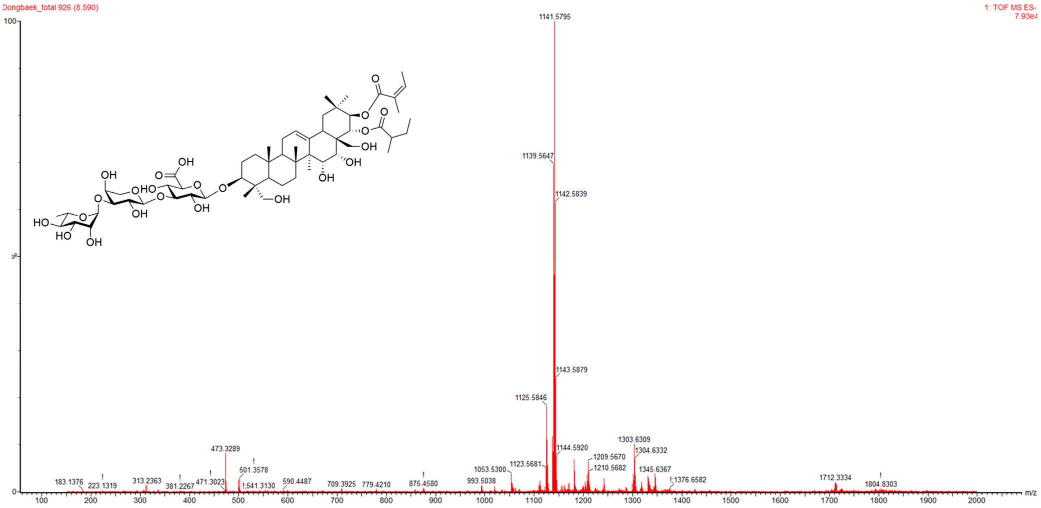

Compound 5

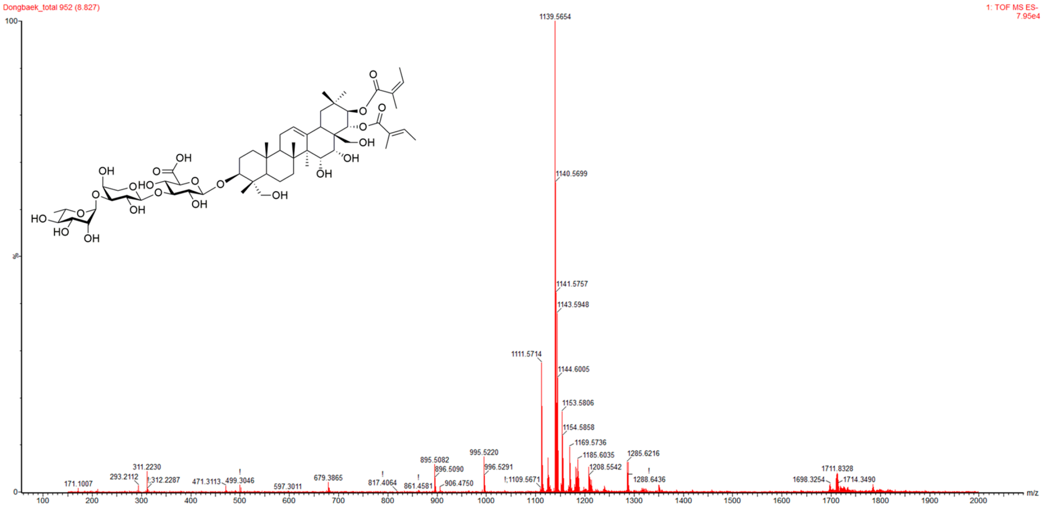

Compound 6

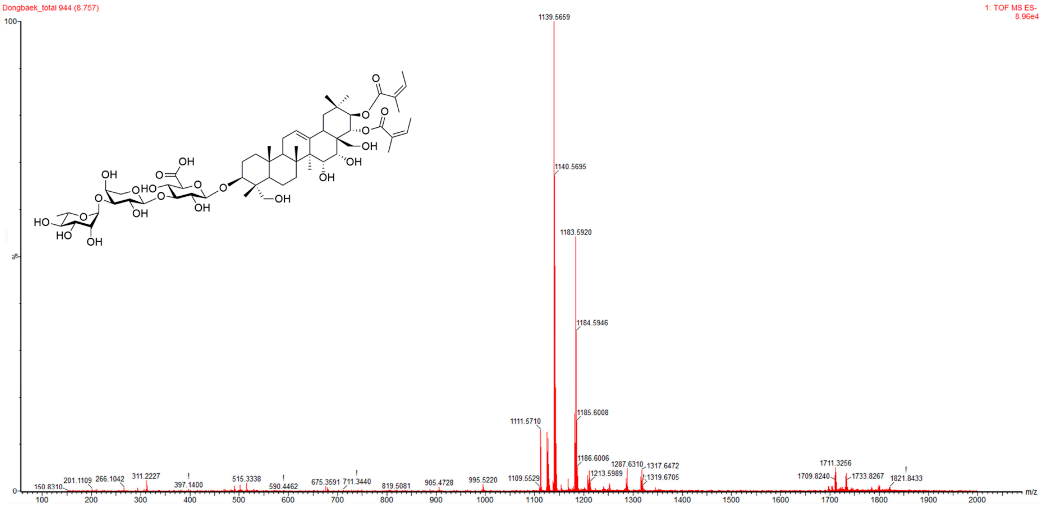

Compound 7

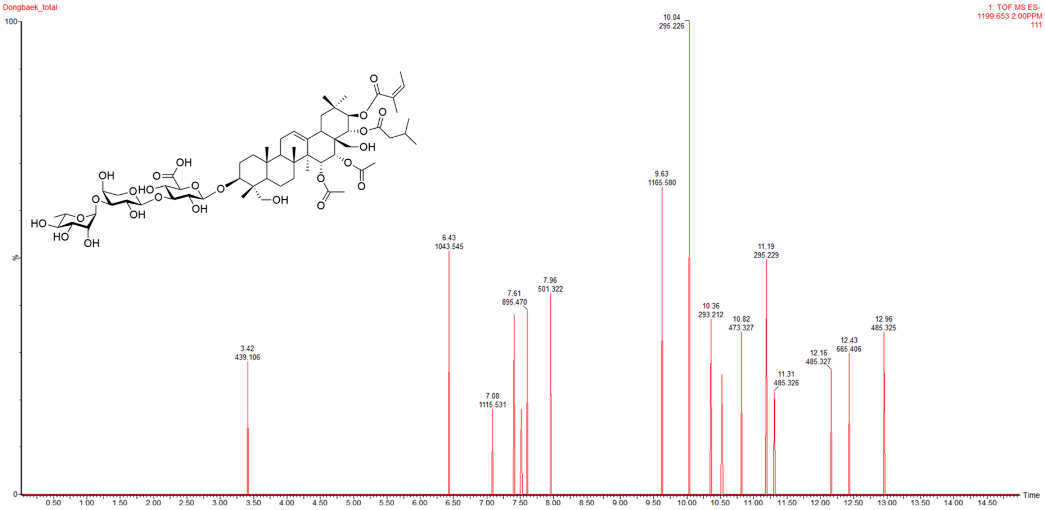

Compound 8

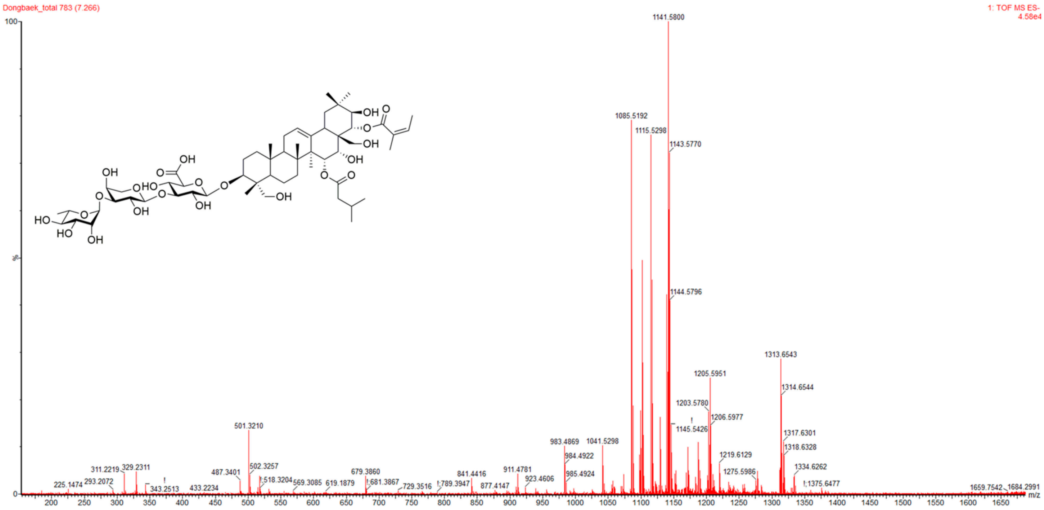

Compound 9

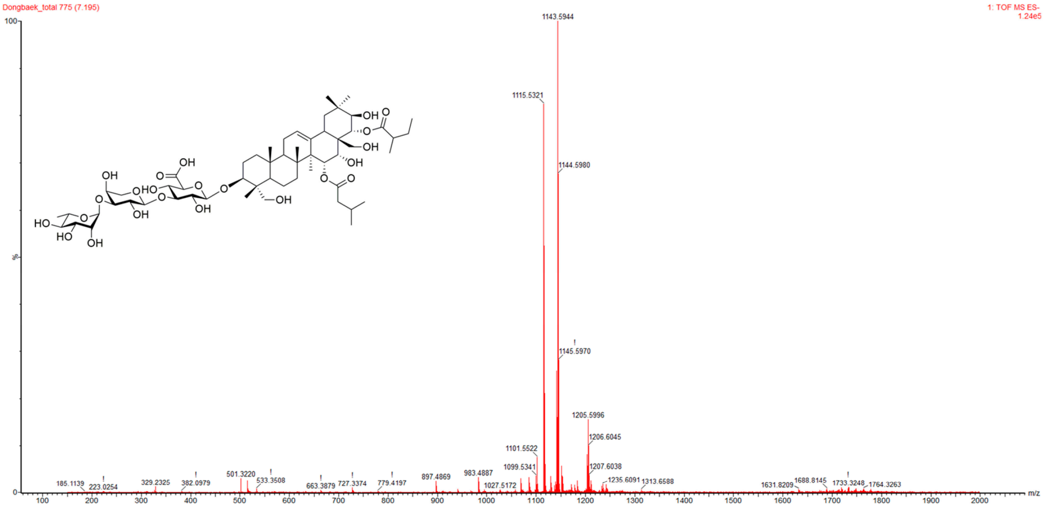

Compound 10

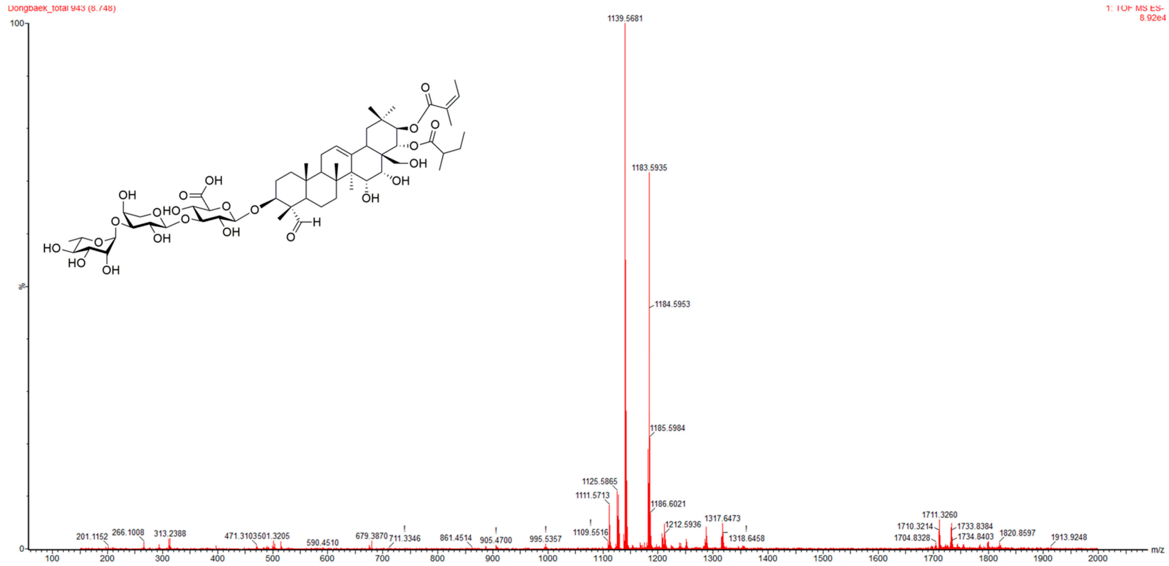

Compound 11

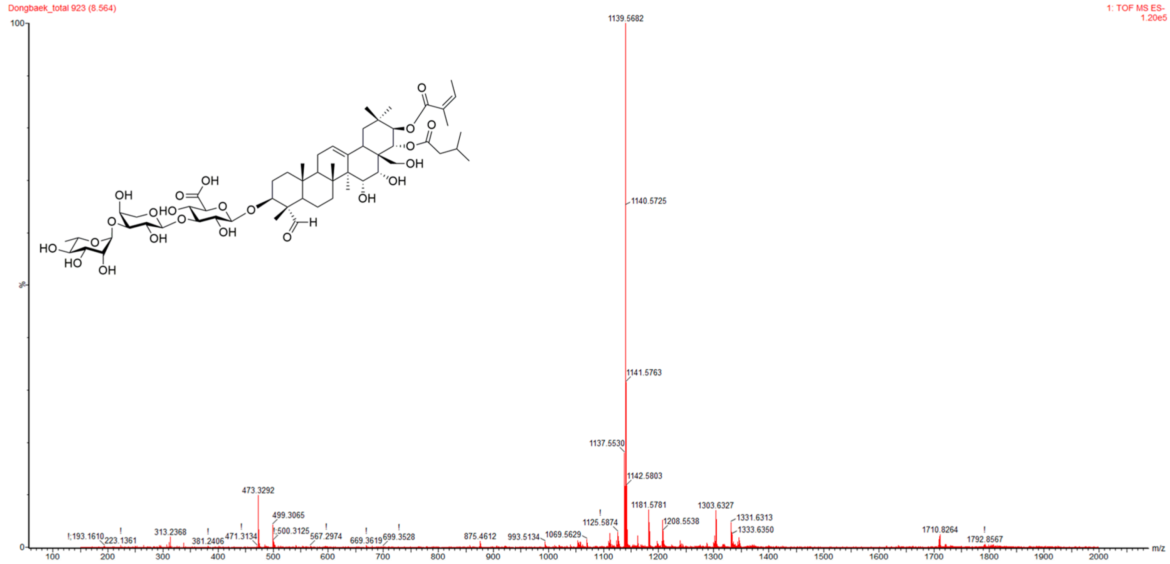

Supplementary Figure S2. The MS spectral data and structures of eleven compounds (1-11) in CJRE
